# Chemical properties and toxicity of soils contaminated by mining activity

**DOI:** 10.1007/s10646-014-1266-y

**Published:** 2014-06-06

**Authors:** Baran Agnieszka, Czech Tomasz, Wieczorek Jerzy

**Affiliations:** Department of Agricultural and Environmental Chemistry, University of Agriculture in Krakow, Al. Mickiewicza 21, 31-120 Krakόw, Poland

**Keywords:** Soil, Heavy metals, Toxicity, Phytoxkit, Microtox

## Abstract

This research is aimed at assessing the total content and soluble forms of metals (zinc, lead and cadmium) and toxicity of soils subjected to strong human pressure associated with mining of zinc and lead ores. The research area lay in the neighbourhood of the Bolesław Mine and Metallurgical Plant in Bukowno (Poland). The study obtained total cadmium concentration between 0.29 and 51.91 mg, zinc between 7.90 and 3,614 mg, and that of lead between 28.4 and 6844 mg kg^−1^ of soil d.m. The solubility of the heavy metals in 1 mol dm^−3^ NH_4_NO_3_ was 1–49 % for zinc, 5–45 % for cadmium, and <1–10 % for lead. In 1 mol HCl dm^−3^, the solubility of the studied metals was much higher and obtained values depending on the collection site, from 45 to 92 % for zinc, from 74 to 99 %, and from 79 to 99 % for lead. The lower solubility of the heavy metals in 1 mol dm^−3^ NH_4_NO_3_ than 1 mol HCl dm^−3^ is connected with that, the ammonium nitrate has low extraction power, and it is used in determining the bioavailable (active) form of heavy metals. Toxicity assessment of the soil samples was performed using two tests, Phytotoxkit and Microtox^®^. Germination index values were between 22 and 75 % for *Sinapis alba*, between 28 and 100 % for *Lepidium sativum*, and between 10 and 28 % for *Sorghum saccharatum*. Depending on the studied soil sample, *Vibrio fischeri* luminescence inhibition was 20–96 %. The sensitivity of the test organisms formed the following series: *S. saccharatum* > *S. alba* = *V. fischeri* > *L. sativum.* Significant positive correlations (*p* ≤ 0.05) of the total and soluble contents of the metals with luminescence inhibition in *V. fischeri* and root growth inhibition in *S. saccharatum* were found. The general trend observed was an increase in metal toxicity measured by the biotest with increasing available metal contents in soils. All the soil samples were classified into toxicity class III, which means that they are toxic and present severe danger. Biotest are a good complement to chemical analyses in the assessment of quality of soils as well as in properly managing them.

## Introduction

Mining and processing of zinc–lead ores are activities that can enrich surrounding area with heavy metals (Muhammad et al. 2011). In many regions of the world where zinc and lead ores are mined and processed, zinc, cadmium, lead, arsenic, thallium, and iron are transported to the environment (Lee and Kao 2004; Degeyse et al. 2004; Loureiro et al. 2005; Aydinalp and Marinova 2003). The adverse effects of heavy metals in these areas are connected with their transfer in the trophic chain, from soil through plants to animal and human (Torres and Johnson 2001; Wolterbeek and Verburg 2001). Most human exposure to metals is associated with contaminated groundwater and soils (Aelion and Davis 2007). Currently, the pollution of soil by heavy metals is evaluated with an assessment, which involves a chemical analysis of the concentration of heavy metals. Soil monitoring in Poland is based mainly on the maximum permissible contents which are given in the Regulation of the Minister of Environment (2002). However, chemical monitoring alone does not always reveal the real threat connected with the presence of heavy metals in the soil (Alvarenga et al. 2008; Venditti et al. 2000). Single and sequential extraction protocols have been designed to predict both the retention and release of metals in soils (Zhang et al. 2013). However, these methods are empirical and can only estimate the potential availability of metals for organisms (Sahuquillo et al. 2003). For this reason, chemical analyses are seldom suited for the evaluation of potential ecological risks, since they do not take into account the possible combined effects of different contaminants as well as their bioavailability (Boularbah et al. 2006a; Alvarenga et al. 2008). Biotests are useful tools which application enables a complete assessment of risk (Römbke et al. 2005; Boularbah et al. 2006a; Płaza et al. 2010; Garcia-Lorenzo et al. 2009; Põlluma et al. 2004). Many authors emphasize that biotests are a good complement to chemical analyses in procedures to assess the quality assessment of soils (Põlluma et al. 2004; Płaza et al. 2010). Due to the fact that organisms differ in sensitivity to various substances, it is essential to select appropriate organisms for test, searching for different taxonomic groups and candidates to represent different links of the trophic chain (Mankiewicz-Boczek et al. 2008; Plaza et al. 2010). Many authors’ studies have shown the suitability of two biotests, Phytotoxkit (plants test) and Microtox^®^ (bacterial test), in the assessment of toxicity of bottom sediments, composts, sewage sludge, and soils (Boularbah et al. 2006a, b; Czerniawska-Kusza and Kusza 2011; Kopeć et al. 2013; Mamindy-Pajany et al. 2011; Loureiro et al. 2005; Dubova and Zarina 2004). Plants are important components of ecosystems; they are the primary food producers and therefore it is important to identify the magnitude of the toxic effects on these organisms (Garcia-Lorenzo et al. 2009). Bacteria play a role as decomposers in environment, and hence there is justification for their inclusion in a battery of tests for assessing soil toxicity (Kahru et al. 2005).

The aims of this study were as follows: (1) to investigated total content and soluble forms of metals (zinc, lead and cadmium) in soil collected in the zinc and lead ore mining and metallurgy area; (2) to use two biotests (Phytotoxkit, Microtox^®^) to evaluate the toxicity of soil, (3) moreover, a possible relationship between the observed toxicity and content of heavy metals was studied. The obtained information may provide a better understand the limitations and benefits of chemical and ecotoxicological methods for conducting the environmental risk assessment of heavy metal polluted soils.

## Materials and methods

### Characteristics of the research area

**Fig. 1:**
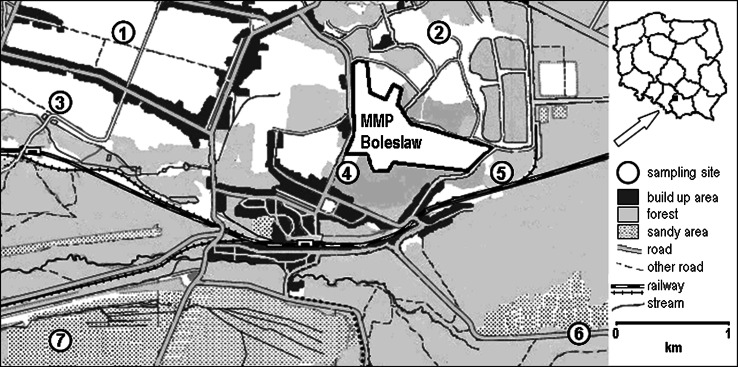
Sampling sites. *MMP Bolesław* Bolesław mine and metallurgical plant

The research area lies in the neighbourhood of the Bolesław Mine and Metallurgical Plant in Bukowno (Fig. 1). This plant is located in the Olkusz zinc and lead ore mining and metallurgical area. It is located between two provinces: Małopolska and Silesia in southern Poland. It is an area where high concentrations of heavy metals in soil, plants, and animal organisms have been recorded. The chemical composition of soils in the Olkusz region largely results from the composition of parent rocks which form them. The substratum of the Triassic carbonates of the Olkusz district consists of Cambrian, Ordovician, and Sylurian rocks, which are covered by sequences of Devonian carbonates as well as Lower and Upper Carboniferous carbonates, sandstones, and clays. Ninety-two percent of the zinc-lead mineralization is hosted by ore-bearing dolomites. Compared with other soils in Poland, soils located in that region are distinguished by increased contents of cadmium, lead, zinc, and iron and other trace elements. The trace elements are more concentrated in the soil surface layer, which may be a result of the present and past ways of acquiring and processing Zn–Pb ores (Cabala and Teper 2007). The areas of former mining and processing of zinc-lead ores in the region are characterized by increased concentrations of harmful elements, caused mainly by primitive methods of mining and processing. Unfortunately, most traces of past mining-metallurgical activities have been expunged and now only the high metals content in the soils remains as evidence, presenting a severe danger to organisms living within these places.

### Soil and plant sample collection

Seven soil samples were collected two locations in the research area from two locations: one directly adjacent to the Bolesław Mine and Metallurgical Plant (sampling site 2, 4, 5) and another one further from the plant (sampling site 1, 3, 6, 7), at a distance of 3,000 and 4,000 m (Fig. 1). Soil material was collected with an Eijkelkamp sampler within a 0–10 cm layer in locations determined using GPSMAP 60CSx satellite navigation (maximum accuracy depends on weather and is up to 0.5 m).

### Chemical analyses of the soil samples

The collected soil samples were dried and then sifted through a sieve with a 2 mm mesh. In soil prepared this way, basic soil properties were determined: pH in 1 mol KCl by the potentiometric method; granulometric composition (using the Casagrande method modified by Prószyński); the content of organic carbon using the Tiurin method; and the sorption capacity of soil (Ostrowska et al. 1991). Besides that, total concentrations of cadmium, lead, and zinc along with forms of these elements extracted with 1 mol dm^−3^ HCl and 1 mol dm^−3^ NH_4_NO_3_ in the soil surface layer were determined in all soil samples (Rauret 1998; Pueyo et al. 2004). The extraction of soluble forms of metals from soils was conducted using a static method consisting of a single shaking of soil samples with a solution of 1:10 soil-to-solution ratio for an extraction time of 1 h. The total concentration of metals was determined after digestion in a muffle furnace and after the dilution of ash in a mixture of HNO_3_ and HClO_4_ (2:1 v/v). Cadmium, lead, and zinc were determined with the use of a Jobin–Yvon Ultrace 238 JY Inductively Coupled Plasma Atomic Emission Spectrometer (ICP-AES). An internal standard and a certified reference material, CRM023-050-Trace Metals-Sandy Loam 7 (RT Corporation), were added to each series of determinations in order to verify their precision. Each sample of the soil material was analysed in two replications. If the replications analysis results differed from one another by more than 5 %, another two analyses have to be conducted for that same sample. A Microsoft Excel 2007 spreadsheet and Statistica 10 package were used for analysis and presentation of the obtained results.

The degree of soil pollution with cadmium, zinc and lead was evaluated using the pollution index (PI) (Wei and Yang 2010). The PI value was calculated as a ratio of the metal content in the sample being evaluated to its geochemical background according to the following formula:

$$ PI = C_{n} /B_{n} $$

where *C*_*n*_ concentration of the analysed element, *B*_*n*_ geochemical background for the analysed element

If *PI* < 1 indicates low, 1 ≤ *PI* < 3 average, and *PI* > 3 severe soil pollution with a given metal. The background concentrations were given by Kabata-Pendias and Pendias (2001), in milligrams per kilogram of dry matter, as follow: 40 mg kg^−1^ for Zn, 0.22 mg kg^−1^ for Cd, and 18 mg kg^−1^ for Pb

### Toxicity of the soil samples

The research involved screening tests with consisted non-diluted samples. The toxicity of the soil samples was studied using two biotests: Microtox^®^ and Phytotoxkit. First, using the Microtox^®^, the toxicity was measure on water extracts using *Vibrio fischeri* bacteria, with luminescence measurement in an M 500 Analyser (Microbics Corporation 1992). A characteristic property of *Vibrio* bacteria is the large amount of metabolic energy used for luminescence. Luminescent bacteria emit visible light as an effect of their normal metabolic processes. Any change in metabolism caused by a toxic substance leads to a change in the intensity of the emitted light. Data analysis was performed using the Microtox Omni software and the standard test procedure 81.9 % Screening Test. Water extracted from the soil was prepared by mixing one volume of the soil with four volumes of redistilled water and shaking them mechanically for 24 h (Loureiro et al. 2005). After that time, the samples were spun for 10 min at a speed of 3,500 rpm and filtered. Luminescence was measurement before and after incubation of the bacterial suspension with the studied sample (after 15 min). Toxicity results were expressed as percent effect (PE%). Three replicate samples were tested.

For the second Phytotoxkit biotest was used to determine chronic toxicity of the soil samples on higher plants. That test was conducted on three plant species: *Sorghum saccharatum* (L), *Sinapis alba* (L), and *Lepidium sativum* (L). The measurement parameters were the inhibition of seed germination (IG) and root length inhibition (IR) in the tested soil in comparison with the control soil. The test was conducted in accordance with the procedure recommended by the manufacturer (Phytotoxkit 2004). Percent germination inhibition (IG) and percent root growth inhibition (IR) were calculated according to the following formula:

$$ {\text{IG or IR }} = \, \left[ {\left( {{\text{A }}{-}{\text{ B}}} \right)/{\text{A}}} \right]{ 1}00\% , $$

where A is the mean seed germination or root length in the control, and B is the mean seed germination or root length in the test soil.

Among various phytotoxicity indices based on germination and early growth of higher plants, the germination index (GI) appears to be a good method for assessing the toxicity of bottom sediments, composts, and soils (Beltrami et al. 1999; Emino and Warman 2004; Czerniawska-Kusza and Kusza 2011; Devesa-Rey et al. 2008). GI was calculated according to the formula for the combined evaluation of the two parameters (Beltrami et al. 1999):

$$ {\text{GI}} = \left( {\text{GsLs}} \right)/\left( {\text{GcLc}} \right) 100\% , $$

where Gs and Ls are seed germination (%) and root elongation (mm) for the sample, and Gc and Lc are the corresponding control values. GI values within the range of 90–110 % were classified as showing no effect/non-toxicity, GI values <90 % where classified as inhibition, and GI values >110 % where classified as stimulation (Beltrami et al. 1999; Czerniawska-Kusza and Kusza 2011).

The toxicity classification system developed by Persoone et al. (2003) was used for assessing the toxicity of soils. After determining the percentage effect for each biotest, a soil sample was classified into one of five classes based on the highest toxicity value indicated by at least one test:

- Class I—no severe risk; none of the tests showed a toxic effect; PE < 20 %,
- Class II—low risk; statistically significant PE is shown in at least one test; 20 ≤ PE > 50 %,
- Class III—high risk; PE value = 50 % is indicated or exceeded in at least one test, but the effect level is lower than 100 %,
- Class IV—high severe risk; PE = 100 % in at least one test,
- Class V—very high severe risk; PE = 100 % all tests.

## Results

### Physico-chemical properties of soils

Basic physico-chemical properties of the soils are presented in Tables 1 and 2. The studied soils displayed various granulometric compositions from light sand to sandy loam (Table 1). A high diversity in pH (4.63–8.04) of the studied soils was found. The soils showed acid (sampling sites 6 and 7), slightly acid (sampling site 1), neutral (sampling sites 4 and 5), and alkaline (sampling sites 2 and 3) reactions. The lowest content of organic carbon was found in a soil sample collected from sampling site 7 (0.5 g kg^−1^ d.m), and the highest from sampling site 4 (32.3 g kg^−1^ d.m) (Table 2). The sorptive capacity (T) of the soils was evaluated based on the determination of hydrolytic acidity (Hh) and the total number of alkali cations (S) as well as V %, which is the degree of sorption complex saturation with bases (Table 2). Values of hydrolytic acidity in the studied soils varied from 5.2 (sampling sites 3 and 7) to 57.4 (sampling site 6) mmol (+) kg^−1^ d.m. The S value is the index of sorptive capacity of soil in relation to alkali cations, and it depends on the grain size, content of organic matter and soil acidity. The higher the acidity, the lower the participation of alkali cations in the soil sorptive complex. Values of alkali cations S in the studied soil samples were from 60.6 (sampling site 6) to 563.5 (sampling site 2) mmol (+) kg^–1^ d.m (Table 2). The domination of alkali cations (from 64 to 99 V%) was found in the soil sorptive complex. The highest cation-sorptive capacity was found in a soil sample collected from sampling site 2 (571.5 mmol (+) kg^–1^ d.m.) and the lowest from sampling site 7 (65.8 mmol (+) kg^–1^ d.m.).

**Table 1:** Some soil characteristics of the soils at the seven sampling sites

Sample numbers	Geographical location UTM 34 (m)		Way of use	Granulonietnc composition	% fraction <0.02 mm
	x	y			
1	388061	5571965	Wasteland	sandy loam	22
2	391947	5571978	Young forest	sandy loam	23
3	386987	5570946	Wasteland	sand	9
4	390940	5569996	Grassland	loamy sand	18
5	392910	5569985	Young forest	sand	1
6	394017	5569171	Forest	sand	3
7	387022	5568273	Wasteland	sand	i

**Table 2:** Basic properties of the soils at the seven sampling sites

Sample numbers	pH	Hh^a^	S^b^	T^c^	V^d^	C organic
	KC1	mmol (+) kg^−1^			%	g kg^−1^ d.m.
1	6.38	23.4	158.1	181.5	87	20.2
2	7.40	7.9	563.5	571.5	99	18.2
3	8.04	5.2	539.7	544.9	99	13.4
4	6.78	14.4	505.1	519.5	97	32.3
5	6.72	17.4	123.1	140.5	SS	5.9
6	4.63	57.4	101.7	159.1	64	17.6
7	5.03	5.2	60.6	65.8	92	0.5
Mean	6.4	18.7	293.1	311.8	89.4	15.4
SD	1.2	18.4	229.7	221.8	12.3	10.3

^a^Hydrolytic acidity

^b^Base exchange capacity

^c^Cation exchange capacity

^d^Degree of base cation saturation in the sorption complex

Total cadmium concentration varied between 0.29 mg and 51.91 mg kg^−1^ of soil d.m., total lead concentration between 7.90 and 3,614 mg, and total zinc concentration between 28.4 and 6,844 mg (Table 3). The highest concentration of the studied metals was found in a soil sample collected from sampling site 2 and the lowest from sampling site 7. Apart from the soil sample collected from sampling site 7, very high total concentrations of metals were recorded in the soils. Other authors’ studies have shown that soils in areas of zinc–lead ore mining and metallurgy areas are characterized by very high concentrations of zinc, lead, and cadmium. The litter and surface layer of forest soils in areas of zinc–lead ore mining and metallurgy areas often contain over 10,000 mg Zn, up to 5,000 mg Pb, and up to 100 mg Cd kg^−1^ (Cabała and Teper 2007; Cabała et al. 2008; Degeyse et al. 2004; Loureiro et al. 2005). The calculated values of the pollution index assumed the following values: from 16 to 236 for cadmium, from 9 to 171 for zinc, and from 6 to 201 for lead. They are indicative of heavy metal pollution of the studied soils. However, on assessing the concentration of metals in the soils, based on the maximum permissible values for soils given in the Regulation of the Minister of Environment (2002), it was established that they met the standards for soils of group C (industrial areas, surface mining land in use, transportation areas).

**Table 3:** Heavy metal concentrations in the soils from the 7 sampling sites

Sample numbers	Total metal concentration			Concentration of soluble forms of metals					
				1 mol dm^−3 ^HC1			1 mol dm^−3 ^NH_4_NO_3_		
	Cd	Pb	Zn	Cd	Pb	Zn	Cd	Pb	Zn
	mg kg^−1 ^d.m.								
1	7.15	173.7	697.5	7.65	166.2	402.9	0.41	4.52	34.52
2	51.91	3614	6844	51.60	3575.7	6264	6.16	11.16	179.98
3	3.52	111.4	341.9	3.46	104.9	179.3	0.17	4.68	2.68
4	45.SI	1283	4215	45.01	1008.5	3696	4.57	2.04	335.80
5	5.24	187.1	496.7	3.86	148.8	225.3	1.21	6.65	102.43
6	6.69	266.5	358.3	5.73	263.6	180.6	3.02	26.59	174.54
7	0.29	7.90	28.4	0.08	0.8	3.7	0.03	0.35	1.98
Mean	17.2	806.2	1854	18.29	752.64	1564	2.22	8.00	118.8
SD	21.8	1310	2630	23.41	1288	2450	2.41	8.89	121.4

Apart from information on the total metals concentrations in the soil, knowledge of their easily soluble forms is also useful due to the possibility of mobilizing them from the solid phase and permeation to the environment. It is possible to estimate the actuation of metals soluble in 1 mol HCl dm^−3^ as a result of the acidification of the environment in which they live. However, using ammonium nitrate enables the isolation of a mobile, readily available fraction of heavy metals from soils. Under natural conditions, this fraction may be released into soil solution, presenting a real danger to living organisms. The solubility of the heavy metals in 1 mol dm^−3^ NH_4_NO_3_ with respect to their total concentration was from 1 % (sampling site 3) to 49 % (sampling site 6) for zinc, from 5 % (sampling site 3) to 45 % (sampling site 6) for cadmium, and from <1 % (sampling sites 2 and 4) to 10 % (sampling site 6) for lead (Table 3). In 1 mol HCl dm^−3^, the solubility of the studied metals was much higher and varied, depending on the collection site, from 45 % (sampling site 3) to 92 % (sampling site 2) for zinc, from 74 % (sampling site 5) to 99 % (sampling sites 1 and 2) for cadmium, and from 79 % (sampling site 4) to 99 % (sampling sites 2 and 6) for lead in comparison with their total concentrations in the soils (Table 3). Many authors claim that contrary to using acidic conditions such as to 1 mol HCl dm^−3^, using a 1 mol dm^−3^ NH_4_NO_3_ solution is classified as a solution with low extraction power, and it is used in determining the so-called bioavailable (active) fraction of heavy metals (Pueyo et al. 2004). As expected, this research found much lower amounts of the studied elements in 1 mol dm^−3^ NH_4_NO_3_. In general, hydrochloric acid washes away metals associated with the exchangeable fraction, carbonate fraction, fraction of Fe–Mn oxides, and organic matter fraction. It is commonly acknowledged that lead is the least mobile metal in the environment, whereas zinc and cadmium are among the most mobile metals (Kabata-Pendias and Pendias 2001). Among the metals studied in this research, cadmium was characterized by the highest average solubility in 1 mol HCl dm^−3^, followed by lead and zinc, whereas in the case of 1 mol NH_4_NO_3_ dm^−3^ the highest average solubility was cadmium > zinc > lead. Numerous researches studies have proved that cadmium is very mobile in soil environments and shows potentially high toxicity for living organisms, even at low concentrations (An 2004).

Linear correlation coefficients between individual pairs of metals as well as between the concentration of metals and other physico-chemical properties of soils were also calculated (Table 4). A linear dependency between individual heavy metals in soil may be a result of their geochemical connections, and it can also inform about their mobility and sources of origin (Guo et al. 2012). A strongly positive correlation between total contents of zinc, cadmium, and lead was found in the studied soils. Strong linear correlations between individual pairs of heavy metals confirm that they are of similar origin, usually connected with human activities and particularly with mining as well as the processing of zinc and lead ores. The research also showed a significantly positive correlation between the total concentration of metals and their forms soluble in 1 mol HCl dm^3^ (cadmium, lead, zinc) and in 1 mol NH_4_NO_3_ dm^3^ (cadmium, zinc). The concentration of metals in the studied soils was generally positively correlated with pH, organic carbon content, sorptive capacity, and percentage content of the finest fraction <0.02 mm (Table 4).

**Table 4:** Correlations between heavy metal concentrations and soil properties and their toxicity to the test organisms

Parameters	Total concentration			lmol HCl			1 mol NH_4_NO_3_		
	Cd	Pb	Zn	Cd	Pb	Zn	Cd	Pb	Zn
Total concentration									
Cd	–	0.90**	0.98**						
Pb	0.90**	–	0.97**						
Zn	0.98***	0.97***	–						
1 mol HC1									
Cd	0.98***	089**	0.98**	–	0.86*	0.98**			
Pb	0.86*	0.97***	0.95**	0.86*	–	0.94**			
Zn	0.92***	0.97***	0.98**	0.98*	0.94**				
1 mol NH_4_NO_3_									
Cd	0.92***	0.88***	0.91**	0.91*	0.86*	0.91**	–	0.35	0.83*
Pb	−0.2	0.11	−0.23	−0.04	0.13	−0.02	0.35	–	0.24
Zn	0.78″	0.51	0.65	0.76	0.45	0.65*	0.83	0.24	–
pH	0.38	0.39	0.42	0.39	0.38	0.41	0.16	−0.42	0.20
C-org.	0.67*	0.38	0.54	0.67*	0.33	0.53	0.60	0,12	0.76
T	0.72*	0.65	0.71*	0.73*	0.63	0.71*	0.58	−0.12	0.42
Fraction < 0.02	0.70*	0.65	0.71*	0.72	0.63	0.70*	0.52	−0.18	0.34
IG									
*Sa*	−0.06	−0.15	−0.21	−0.08	−0.17	−0.10	−0.05	−0.21	0.18
*Ls*	0.30	0.01	0.18	0.31	−0.04	0.20	0.10	−0.41	0.31
*Ss*	0.58*	0.76^*^	0.70*	0.58	0.76	0.70	0.42	−0.36	0.05
IR									
*Sa*	−0.33	−0.48	−0.44	−0.35	−0.50	−0.42	−0.13	0.28	0.23
*Ls*	−0.22	−0.37	−0.33	−0.25	−0.39	−0.32	−0.01	0.34	0.33
*Ss*	0.67*	0.59*	0.62*	0.65*	0.57*	0.62*	0.79*	0.43	0.75*
IL									
*Vf*	0.75*	0.85*	0.81*	0.75*	0.85*	0.82*	0.77*	0.21	0.42

Significant at ***p ≤ 0.001; **p ≤ 0.01; *p ≤ 0.05

IG, inhibition of germination; IR, inhibition of roots growth; IL, inhibition of luminesence; *Sa*, *Sinapis alba*; *Ls*, *Lepidium sativum*; *Ss*, *Sorghum saccharatum*; *Vf*, *Vibrio fischeri*

### Soil ecotoxicity

Table 5 presents the results of the toxicity assessment of the soils collected from the region influenced by of Bolesław Mine and Metallurgical Plant in Bukowno. The inhibition of seed germination of the test plants was between 0 and 45 %. Among the test plants, the highest germination inhibition was found in *S. saccharatum* (between 20 and 45 %), and the lowest in *S. alba* (between 0 and 20 %). Among the studied soil samples, the strongest inhibition of *S. saccharatum* seed germination was observed in the case of soil from sampling site 2, while for *L. sativum* the strongest inhibition was observed in soil from sampling site 4, and for *S. alba*, sampling site 5. These soils are located very close to the Bolesław Mine and Metallurgical Plant in Bukowno. The plant root growth inhibition was between −11 and 82 % (Table 5). Similarly to germination, the highest root growth inhibition was found in *S. saccharatum* (60–82 %). *L. sativum* was the plant most resistant to the phytotoxic influence of the studied soils. Root growth inhibition of this plant was between −11 and 70 %. The root growth inhibition of *S. alba* varied from 22 to 74 %. Soils collected from sampling sites 2, 5, and 6 caused the highest root growth inhibition of the test plants (Table 5). Depending on the studied soil sample, *V. fischeri* luminescence inhibition was from 20 to 96 %. The highest luminescence inhibition of *V.* *fischeri* was found in a soil sample collected from sampling site 2, and the lowest in a sample from sampling site 1.

**Table 5:** Percentage toxic effects of the soils, for the test organisms and toxicity classification of the soils

Sample numbers	Inhibition (PE%)								
	Germination			Roots			Luminescence	Toxicity classification and class score %	
	*Sa*	*Ls*	*Ss*	*Sa*	*Ls*	*Ss*	*Vf*		
1	5	10	30	19	−11	62	20	22	III
2	5	10	45	77	0	82	96	33	III
3	0	15	30	46	26	74	61	39	III
4	10	20	30	60	47	81	57	64	III
5	20	5	35	79	70	76	30	50	III
6	5	10	20	74	64	78	4S	44	III
7	10	15	30	57	28	60	78	39	III
Mean	8	12	31	50	32	73	49	–	III
SD	6	5	7	22	31	9	25	–	

*Ls*, *Lepidium sativum*; *Sa*, *Sinapis alba*;*Ss*, *Sorghum saccharatum*; *Vf*, *Vibrio fischeri*

**Fig. 2:**
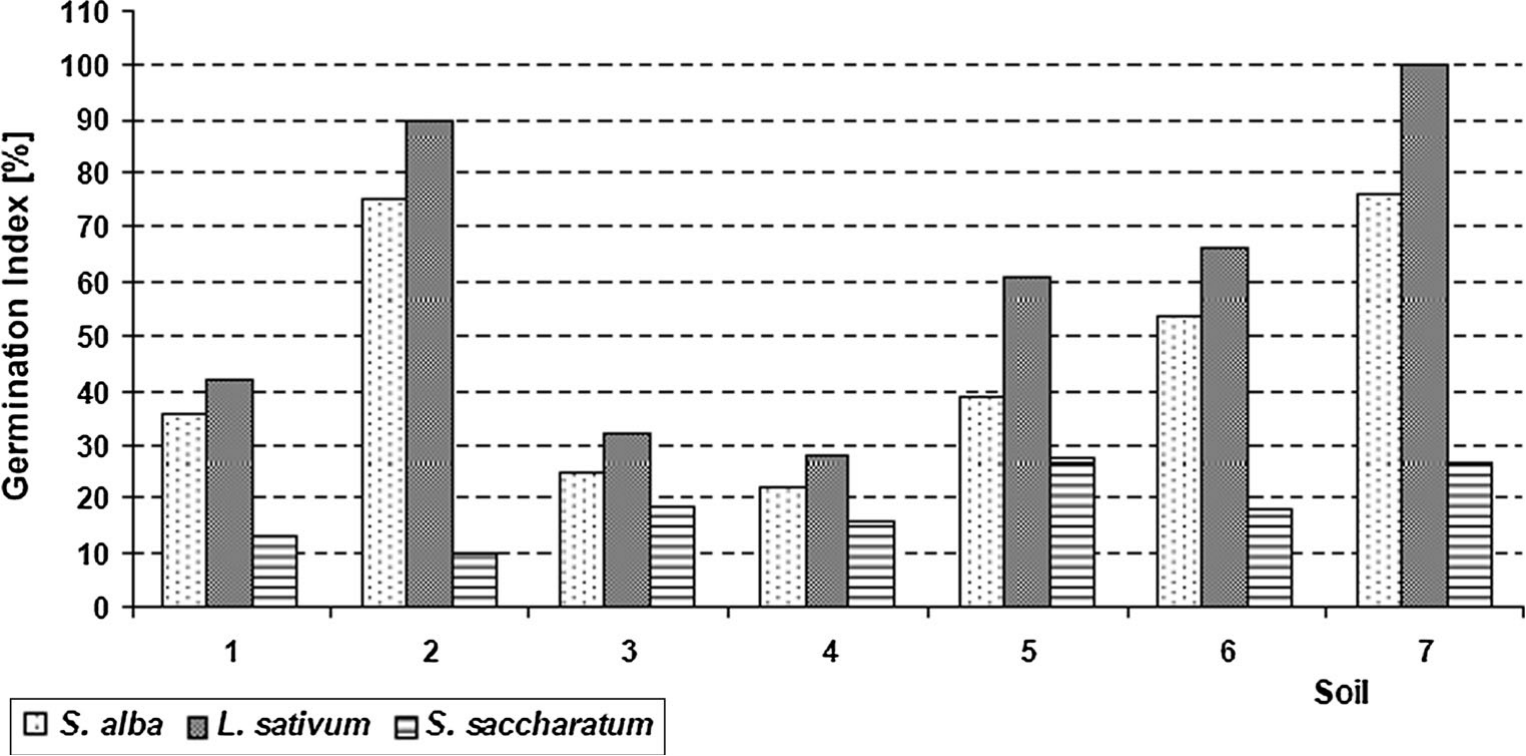
Germination index values (GI) for the three plant species for the seven soil samples

Germination index values for *S. alba* were between 22 % (sampling site 4) and 75 % (sampling site 2) (Fig. 2). This means that all the studied soil samples caused growth inhibition of this plant. The germination index for *L. sativum* varied from 28 % (sampling site 4) to 100 % (sampling site 7). Only soil samples collected from sampling sites 2 and 7 were not toxic for *L. sativum* (GI of 90–110 % indicates no effect/non-toxicity), and the remaining ones caused growth inhibition of the test plant. The lowest GI values were found for *S. saccharatum* (between 10 % and 28 %) (Fig. 2). Similarly to *S. alba*, all the studied soil samples caused the growth inhibition of this plant. According to the toxicity classification system proposed by Persoone et al. (2003) all the soil samples were classified into toxicity class III, which means that they are toxic and present severe danger. The highest toxicity for the test organisms was found in a soil sample collected from sampling site 4, and the lowest in one from sampling site 1. The soil toxicity formed the following series (collection sampling sites): 4 > 5 > 6 > 7 = 3 > 2 > 1 (Table 5).

An analysis of the correlation between the concentration of metals and the results of toxicity to the test organisms was carried out (Table 4). The essential positive values of the correlation coefficients in this research indicate a connection between metal content in soil and toxicity for organisms, whereas negative values (due to the lack of significance) mean that the studied metal did not influence the increase in sample toxicity. A significant positive correlation was found between the total concentration of the metals and luminescence inhibition in *V. fischeri* as well as germination inhibition and root growth inhibition in *S. saccharatum*. Metal forms soluble in 1 mol HCl dm^−3^ (cadmium, lead, zinc) and in 1 mol NH_4_NO_3_ dm^−3^ (cadmium and zinc) were significantly positively correlated (*p* ≤ 0.05) with the luminescence inhibition of *V. fischeri* and with the root growth inhibition of *S. saccharatum* (Table 4). In general, negative correlations with the concentration of metals were found in the case of *S. alba* and *L. sativum*, which may suggest that other compounds which were not studied in this work caused germination inhibition and root growth inhibition of these plants. Research carried out by Płaza et al. (2010) also found a significant positive correlation between the concentration of heavy metals in soils polluted with heavy metals and inhibition of *V. fischeri.* The research also did not find a significant correlation between root growth inhibition of *L. sativum* and the concentration of metals in soils. In turn, research by Garcia-Lorenzo et al. (2009) found a positive correlation between germination inhibition, growth inhibition of the test plants, and the concentration of heavy metals in soils coming from areas of mining activities.

## Discussion

Soils in the Olkusz zinc and lead ore mining and metallurgy area are polluted with cadmium, zinc, and lead as well as thallium, arsenic, and iron, as shown by Cabała and Teper (2007), Cabała et al. (2008), and the presented research. These metals are concentrated mainly in the soil surface layer, which, because of its environmental functions, has a significant influence on the entire biosphere. The high metals concentration in the soils of the studied region are highly variable, which is confirmed by the high standard deviation (Table 3). Research of Trafas et al. (2006) proves that in the Bolesław region, even over a small area (50 × 50 m), the diversity of zinc and lead contents is high. The high variation in the metals concentration in the studied soils is connected with the occurrence of metalliferous minerals whose accumulations depend on natural and anthropogenic factors. The most important of these factors include: geological structure and erosion of shallow ore-bearing Triassic formations; historical mining as well as processing of zinc and lead ores, which are responsible for the surface deposition of waste rich in zinc, lead and cadmium; the emission of metal-rich dust from the zinc works; the high emission of industrial dust from the Upper Silesian Industrial Region as well as the eolian redeposition of zinc-lead-iron minerals from above ground landfills designed for postflotation and post metallurgical waste. In the studied soils, zinc, lead and cadmium most often occurred in the ratio of 100:25:1 (sampling sites 1, 3, 4, 5, 7), which corresponds to ratios determined in zinc-lead ores (Cabala and Teper 2007; Cabala et al. 2008). Based on that it can be concluded that the metals content in the studied soils is connected with the same mineral complexes as the ones that occur in primary or oxidized ores as well as in postflotation waste created as a consequence of their processing. In the studied region, the level of heavy metals concentration in the soils depended also on the distance from the sources of pollution. Changes in the metals concentration in the research area indicate that the content decreases along with the distance from the landfills designed for postflotation waste and waste from zinc smelter. Zinc, cadmium and lead concentrations in soils were highest close to the landfill and the works (sampling sites 2 and 4). Zinc concentration reached, respectively, 6,844 and 4,215 mg, 3,614 and 1,283 mg (lead), and 51.91 and 45.81 mg (cadmium) kg^−1^ of soil. Krzaklewski et al. (2004) obtained similar results; in forest soils neighbouring waste landfills they determined the zinc concentration to be up to 10,638 mg, lead concentration up to 2,696 mg, and cadmium concentration up to 55 mg kg^−1^ of soil.

Many research studies suggest that soil reaction, content of organic matter, and content of the finest fraction may influence the mobility of metals in the environment (Venditti et al. 2000). It was proved that solubility (and consequently bioavailability) of heavy metals increases at low soil pH (acid and very acid soils) (Venditti et al. 2000). In the research study the positive correlation between pH and the concentration of heavy metals in the soils does not confirm the above relation (Table 4). As already mentioned, the studied area is exposed to the direct influence of industrial activities connected with mining and the processing of zinc–lead ores. These ores can be found in ore-bearing dolomites, which are a source of not only zinc, lead, and cadmium, but also calcium and magnesium carbonate, which in turn have an alkaline effect on the environment, which has a beneficial influence on bonding metals in stable carbonate minerals (Cabała et al. 2008). However, it is important to remember that despite a significant influence of the reaction of the substratum on the assimilability of heavy metals by soil organisms, the total concentration of an element has the greatest influence on its bioavailability and toxicity (Favas et al. 2011), which is confirmed in this research. The total metal concentration in soil samples correlated in a significantly positive way with the response of the test organisms (S*. saccharatum* and *V. fischeri*) (Table 4).

**Fig. 3:**
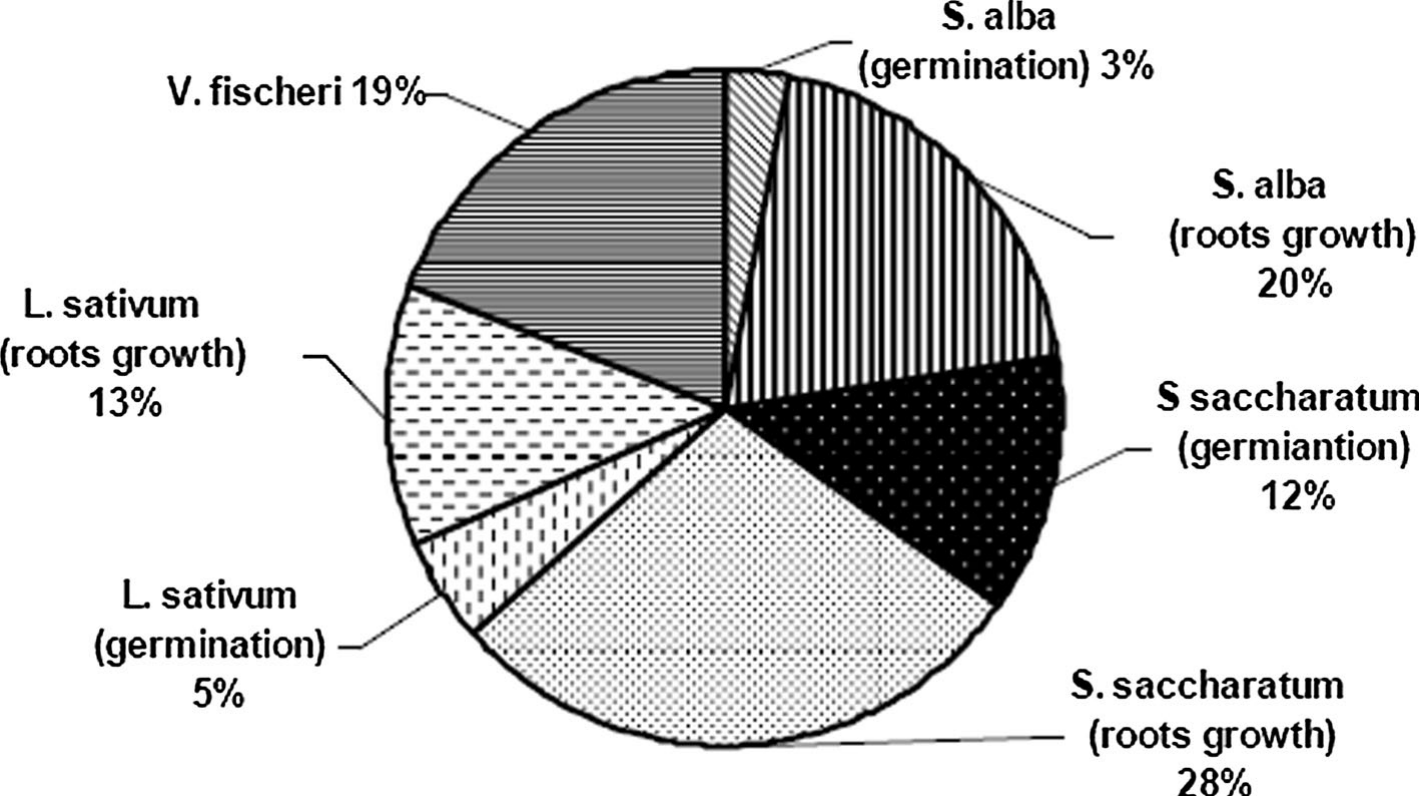
Percentage of toxic response for each applied biotest as the percentage of the total

The quality of soils is generally assessed based on physico-chemical indices, without taking microbiological and ecotoxicological parameters into consideration. The total concentration metals of metals in soils gives little information on their mobility and toxicity. Furthermore, chemical fractionation methods using several extractants do not give adequate information about metal bioavailability for all the metals present in a multi-contaminated soil (Boularbah et al. 1996; Boularbah et al. 2006b; Plaza et al. 2010). Many authors have shown that biotests give a general indication of metal bioavailability in soils and are recommended for the assessment of ecological risks in soil (Boularbah et al. 2006a; Kahru et al. 2005; Zhang et al. 2013). The analysis of results obtained from the conducted research indicates that it seems necessary, however, to apply a battery of bioassays that uses organisms from different trophic levels and with different sensitivity to substances present in soil. It is important that each species and test procedure have their own sensitive pattern to toxicants, because one single species is not sensitive to all chemicals (Matejczyk et al. 2011). In the research, the test organisms were exposed to substances dissolved in water (Microtox) as well to substances absorbed on the surface of solid particles (Phytotoxkit). It is important because heavy metals undergo sorption on non-organic and organic particles and are available only during direct contact. Other authors’ works also drew attention to this aspect and to the selection of test organisms (Płaza et al. 2010; Matejczyk et al. 2011). The research showed that soil samples are toxic, but the used organisms showed different sensitivity to heavy metals occurring in the soils (Table 5; Fig. 3). On estimating the sensitivity of the performed biotests, the highest number of toxic responses was recorded by Phytotoxkit towards *S. saccharatum* and showed 29 % root growth inhibition (Fig. 3). This result on soil samples is in agreement with similar findings by Czerniawska-Kusza et al. (2006), Czarniawska-Kusza and Kusza (2011), and Mamindy-Pajany et al. (2011), who reported that *S. saccharatum* is the most sensitive species in the identification of phytotoxic sediment samples compared to *L. sativum* and *S. alba*. Among all the test organisms, the lowest sensitivity was observed in the case of *L. sativum*. The sensitivity of the test organisms formed the following series: *S. saccharatum* > *S. alba* = *V. fischeri* > *L. sativum* (Fig. 3). In the papers of Mankiewicz-Boczek et al. (2008) and Kopeć et al. (2013), the number of toxic responses was higher in the chronic Phytotoxkit test with higher plants than in the Microtox^®^ test with bacteria *V. fischeri*. Loureiro et al. (2005), using Microtox^®^ to evaluate the toxicity of two mine soils, found that *V. fischeri* was more sensitive to eluates from soil with low heavy metal concentration than eluates from soil with high heavy metal concentration. Alvarenga et al. (2008) found that some tests—earthworm mortality, seed germination, and bacterial luminescence—were less sensitive to mine-contaminated soils, whereas plant growth and *D. magna* immobilization tests were much more sensitive.

## Conclusion

To sum up, biotests carried out on soils collected in the area of zinc and lead ore mining and metallurgy showed that they are toxic for the test organisms. The general trend observed was an increase in metal toxicity measured by the biotest with increasing available metal contents in soils. These soils were classified into toxicity class III. At the same time, their toxicity was higher in soil samples collected at the shortest distance from the Mine and Metallurgical Plant in Bukowno. The direct biotest (*L. sativum*, *S. alba*, *S. saccharatum*), which evaluate habitat function, as well as the aquatic test (*V. fischeri*), which evaluate soil retention function, proved to be adequate to assess the quality of these mine-contaminated soils. Due to their specificity, ecotoxicological tests constitute a good complement to chemical analyses in the assessment of quality of soils as well as in properly managing them.
